# Quantitative Decomposition of Dynamics of Mathematical Cell Models: Method and Application to Ventricular Myocyte Models

**DOI:** 10.1371/journal.pone.0124970

**Published:** 2015-06-19

**Authors:** Takao Shimayoshi, Chae Young Cha, Akira Amano

**Affiliations:** 1 Graduate School of Informatics, Kyoto University, Kyoto, Japan; 2 Oxford Centre for Diabetes, Endocrinology and Metabolism, University of Oxford, Oxford, United Kingdom; 3 College of Life Sciences, Ritsumeikan University, Kusatsu, Shiga, Japan; University of Erlangen-Nuremberg, GERMANY

## Abstract

Mathematical cell models are effective tools to understand cellular physiological functions precisely. For detailed analysis of model dynamics in order to investigate how much each component affects cellular behaviour, mathematical approaches are essential. This article presents a numerical analysis technique, which is applicable to any complicated cell model formulated as a system of ordinary differential equations, to quantitatively evaluate contributions of respective model components to the model dynamics in the intact situation. The present technique employs a novel mathematical index for decomposed dynamics with respect to each differential variable, along with a concept named instantaneous equilibrium point, which represents the trend of a model variable at some instant. This article also illustrates applications of the method to comprehensive myocardial cell models for analysing insights into the mechanisms of action potential generation and calcium transient. The analysis results exhibit quantitative contributions of individual channel gating mechanisms and ion exchanger activities to membrane repolarization and of calcium fluxes and buffers to raising and descending of the cytosolic calcium level. These analyses quantitatively explicate principle of the model, which leads to a better understanding of cellular dynamics.

## Introduction

Mathematical modelling has been an effective method in physiology for precise and comprehensive understanding of the dynamic behaviour of cells. A number of mathematical cell models have been developed, and recent models of cardiac cells [1–5] have been more detailed and thereby complicated by including multiple cellular functions to explain new experimental findings. Conventionally, these models have been used to simulate wet experiments. In contrast with wet experiments, an accurate and more complete set of experimental data can be obtained by numerical simulation. Additionally, mathematical models enable simulation experiments that are otherwise impracticable, such as a pure and complete blockade of an ion channel or a perfect control of the intracellular composition. Despite the success of simulation, such conventional simulation is insufficient to achieve the full potential of mathematical cell models. Since the whole mechanisms of each model dynamics are explicitly defined in mathematical expressions, models potentially enable quantitative clarification of their detailed behaviour, which leads to a better understanding of cellular dynamics.

Each of mathematical cell models is generally formulated as a system of ordinary differential equations (ODE) with respect to time. The ODE model variables interact with each other either directly or indirectly and vary simultaneously. In order to elucidate the causes and results of this interaction, inspection of model equations is essential but difficult for detailed models due to complicated interdependences of variables. To overcome this difficulty, mathematical approaches are required. One such approach applicable to mathematical cell models is bifurcation analysis, which is used to investigate qualitative changes in a system of equations by smooth changes in parameter values. More specifically, the bifurcation analysis can determine whether a model converges, diverges, or oscillates depending on the parameter values. For instance, Kurata and his collaborators [6–12] have applied the bifurcation analysis to mathematical models for understanding the oscillatory phenomena in ventricular and sinoatrial node cells. The singular perturbation method of asymptotic analysis is a method for inspection of the dynamic behaviour of mathematical models. In this method, variables are divided into fast and slow ones, and steady states of a model in regarding the slow variables as parameters are traced in time. Analysis based on this method can explain dynamic change in characteristics, e.g. membrane excitability of cardiac cells [13–16]. These methods can answer why a model has its behaviour.

Another fundamental question in model dynamics is how much each model component affects the model behaviour. In physiological experiments, the most conventional approach for examining contribution of a cellular component is activation or inhibition of a target function using agonists, blockers or knockout of the corresponding gene. The same kinds of methods have been also applied to many simulation studies by altering the corresponding parameter values. However, the interpretation of results of these methods for estimating contribution of a component in physiological condition is extremely difficult in most cases. Since a modification to a component secondarily causes changes in other components which also affect the target function, the resultant change in the function cannot be considered as a sole effect of the modulated component but a mixed effect of the other components. To overcome this difficulty, Clewley et al. [17, 18] have developed ‘dominant scale method’, and Cha et al. [19] ‘lead potential analysis’. However, their methods are limited to analyses of cellular membrane potential.

In this study, a numerical method is introduced for quantitatively decomposing dynamics of mathematical cell models. This method is applicable to analysis of every model variables, and able to evaluate contributions of individual model components to the dynamics of a variable. Firstly, the mathematical definition of the proposed method is presented in this article. Then, applications of the method to action potential and calcium transient of ventricular myocyte models are illustrated.

## Method

This section provides the mathematical definition of a novel index for decomposed dynamics of an object variable in an ODE model, following to introduction of a concept ‘instantaneous equilibrium point.’ For a time-dependent variable *v*, *v* ^(*t*)^ denotes the value of *v* at *t*, and $\dot{v}$ denotes the first derivative of *v* with respect to *t*, i.e., *dv*/*dt*. A variable *v* is called a differential variable if $\dot{v}$ is explicitly defined in a model.

Consider the dynamics of an object differential variable *x*. Let the derivative of *x*

$$\begin{array}{c} \dot{x}=f\left(x,y\right), \end{array}$$ (1)

where **y** is a vector of all the other differential variables.

By introducing an auxiliary time-dependent function ${\hat{f}}^{\left(t\right)}$ such that ${\hat{f}}^{\left(t\right)}(x)=f(x,{\text{y}}^{\left(t\right)})$ at *t* for any value of *x*, for simplicity, Eq 1 at a certain time *t*_*c*_ can be written as

$$\begin{array}{c} \dot{x}={\hat{f}}^{\left(t_{c}\right)}\left(x\right)=f\left(x,y^{\left(t_{c}\right)}\right). \end{array}$$ (2)

By linearizing Eq 2 with the first-order Taylor series of ${\hat{f}}^{\left(t_{c}\right)}(x)$ at *x* ^(*t*_*c*_)^,

$$\begin{array}{c} \dot{x}\;\approx \;{\dot{x}}^{\left(t_{c}\right)}+f_{x}^{\left(t_{c}\right)}\;\cdot \;(x-x^{\left(t_{c}\right)})\;=\;-f_{x}^{\left(t_{c}\right)}\;\cdot \;({\bar{x}}^{\left(t_{c}\right)}-x), \end{array}$$ (3)

$$\begin{array}{c} \bar{x}≜x-\dot{x}/f_{x}, \end{array}$$ (4)

where $f_{x}^{\left(t_{c}\right)}$ stands for the partial derivative of *f* with respect to *x* at (*x* ^(*t*_*c*_)^, **y** ^(*t*_*c*_)^). Eq 3 expresses the tangent line to ${\hat{f}}^{\left(t_{c}\right)}(x)$ at *x* ^(*t*_*c*_)^, or to *f*(*x*, **y**) at the current value of *x* in assuming that **y** is constant with its value at *t*_*c*_. The x-intercept of the tangent line is ${\bar{x}}^{\left(t_{c}\right)}$. Consequently, the linearized Eq 1 at *t*_*c*_ with respect to *x* is obtained:

$$\begin{array}{c} \dot{x}=-f_{x}^{\left(t_{c}\right)}\;\cdot \;({\bar{x}}^{\left(t_{c}\right)}-x). \end{array}$$ (5)

Thus, ${\bar{x}}^{\left(t_{c}\right)}$ is the fixed point of Eq 5, which is stable if $f_{x}^{\left(t_{c}\right)}$ is negative or unstable if positive. Namely, *x* is naively attracted to or repelled from ${\bar{x}}^{\left(t_{c}\right)}$. Hence, $\bar{x}$ is referred to as the *instantaneous equilibrium point* of *x*, which represents the trend in *x* at some instant. Since $\bar{x}$ is time-dependent, the orbit of *x* in Eq 1 is additionally affected by temporal change in $\bar{x}$. Therefore, temporal change in $\bar{x}$ can be regarded as representing active dynamics of *x*.

To decompose the active dynamics of *x*, consider an effect of temporal change in a variable *y* of **y** on a variation in $\bar{x}$ at a certain moment. A sensitivity *k*_*x*,*y*_ of the rate of change in $\bar{x}$ to the rate of change in *y* is mathematically expressed as

$$\begin{array}{c} k_{x,y}=\frac{\partial \dot{\bar{x}}}{\partial \dot{y}}=\frac{\partial \bar{x}}{\partial y}=\frac{f_{xy}\;\dot{x}-f_{x}\;f_{y}}{{\left(f_{x}\right)}^{2}}, \end{array}$$ (6)

where *f*_*xy*_ stands for the second-order mixed derivative of *f* with respect to *x* and *y*, and *f*_*y*_ for the partial derivative of *f* with respect to *y*. Here, *y* *dynamic of* *x* is defined by the sensitivity *k*_*x*,*y*_ weighted by $\dot{y}$, the current rate of change in *y* at the moment:

$$\begin{array}{c} c_{x,y}≜k_{x,y}\;\dot{y}=\frac{\partial \dot{\bar{x}}}{\partial \dot{y}}\;\dot{y}=\frac{\partial \bar{x}}{\partial y}\;\dot{y}=\frac{f_{xy}\;\dot{x}-f_{x}\;f_{y}}{{\left(f_{x}\right)}^{2}}\;\dot{y}. \end{array}$$ (7)

Similarly, *x* *dynamic* of *x* itself is defined:

$$\begin{array}{c} c_{x,x}≜\frac{\partial \bar{x}}{\partial x}\dot{x}=\frac{f_{xx}}{{\left(f_{x}\right)}^{2}}\;{\dot{x}}^{2}, \end{array}$$ (8)

where *f*_*xx*_ stands for the second-order partial derivative of *f* with respect to *x*.

For the chain rule,

$$\begin{array}{c} \dot{\bar{x}}=\frac{\partial \bar{x}}{\partial x}\dot{x}+\sum_{y\in y}\frac{\partial \bar{x}}{\partial y}\;\dot{y}=c_{x,x}+\sum_{y\in y}c_{x,y}. \end{array}$$ (9)

Consequently, *c*_*x*,*y*_ is $\dot{y}$ component of the rate of change in $\bar{x}$, that is, the decomposed dynamics of *x*. The sign of *c*_*x*,*y*_, which is determined by the both signs of *k*_*x*,*y*_ and $\dot{y}$, indicates whether a temporal change in *y* induces *x* to increase in the positive case or decrease in the negative case. Note that *c*_*x*,*x*_ represents an effect of the nonlinearity of $\hat{f}(x)$.

## Application

### Models and Methods

The present method is demonstrated using the two mathematical ventricular myocyte models. One is a guinea pig model proposed by Takeuchi et al. [1], which represents membrane excitation, ion homeostasis, excitation-contraction coupling, volume regulation, and the balance of ATP production and consumption. This model formulates the spermine block and the magnesium block of *I*_K1_, which is a major outward current of ventricular myocytes. In the original model, while the open probability *y* of the spermine block is expressed as a differential equation, the open probability *f*_*O*_ of the magnesium block is defined as a steady-state expression. For that expression a differential equation is substituted in this application (see S1 Appendix), in order to evaluate the contributions of *I*_K1_ through both the magnesium and spermine blocks. As a result, the number of differential variables of the modified model is 51. The other is a human ventricular myocyte model published by Priebe and Beuckelmann [5], which includes action potential generation and calcium dynamics. The number of its differential variables is 22.

A simulation program is coded in C to invoke an ODE solver, CVODE (Lawrence Livermore National Laboratory, Livermore, CA), in which backward differentiation formulae are used and the time step is adaptively controlled. The first-order partial derivatives of the derivative functions of differential variables are obtained with automatic formula differentiation. The second-order partial derivatives are calculated with numerical differentiation. The stimulation protocol applied to each model follows the corresponding original paper [1, 5]. For the Takeuchi model, the stimulation current is injected for 2 ms at 50 ms after the start of the simulation. For the Priebe model, the stimulation is applied for 3 ms at 0 ms. The values of parameters and initial values of variables used in the simulation are identical to the original models.

**Fig 1:**
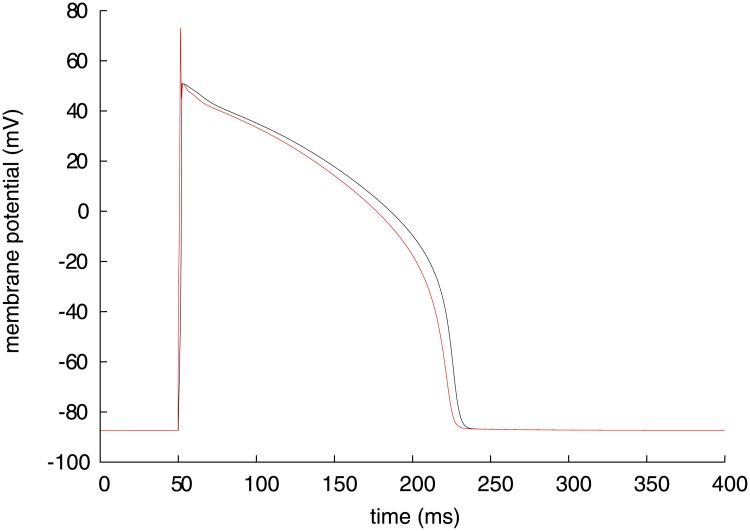
Time course of membrane potential (black) and its instantaneous equilibrium point (red) of Takeuchi model.

**Fig 2:**
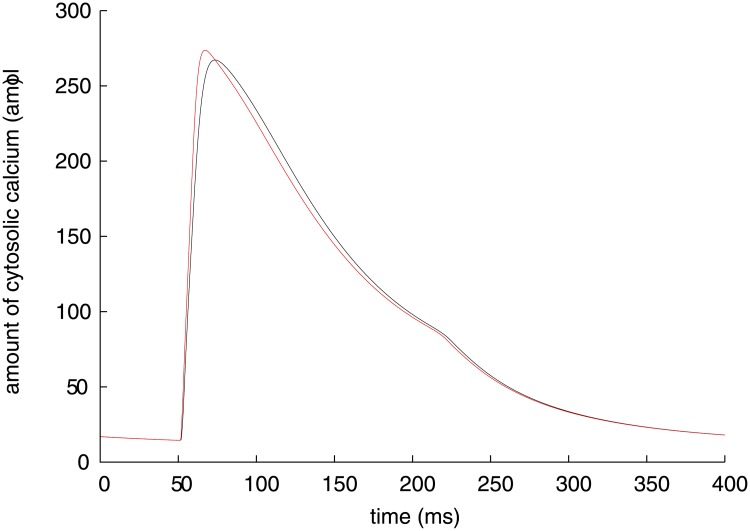
Time course of amount of cytosolic calcium (black) and its instantaneous equilibrium point (red) of Takeuchi model.

**Fig 3:**
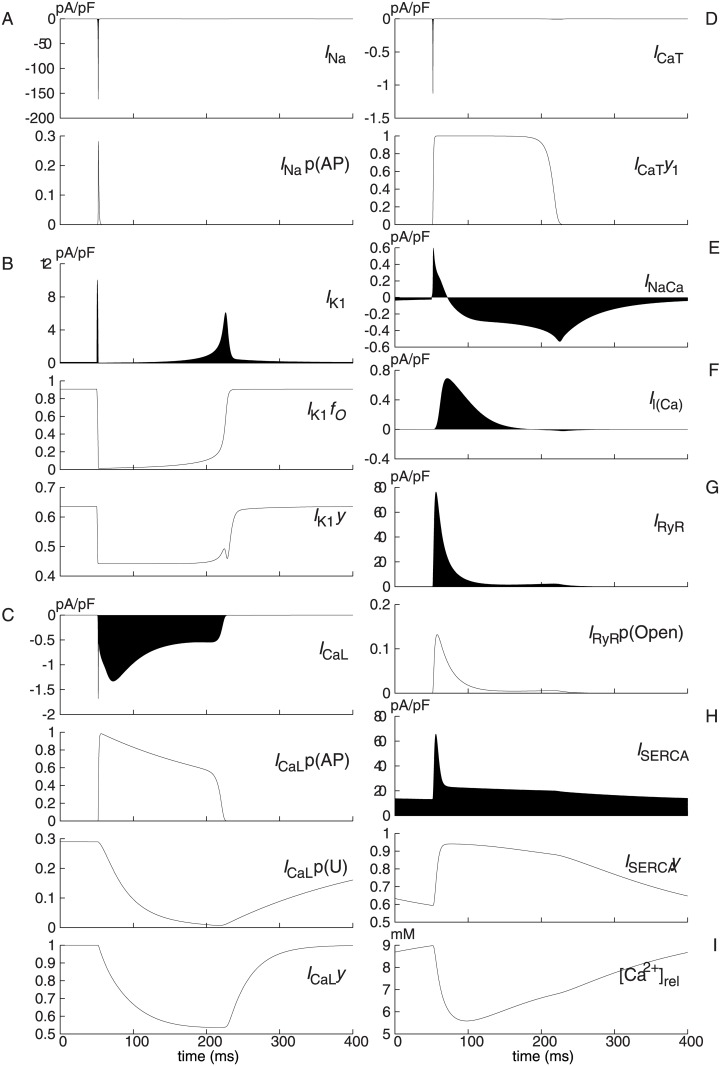
Major currents and differential variables simulated using Takeuchi model. (A) *I*_Na_, fast sodium current; *I*_Na_ p(AP), open probability of *I*_Na_ voltage gate. (B) *I*_K1_, inward rectifier potassium current; *I*_K1_ *f*_O_, open probability of *I*_K1_ magnesium gate; *I*_K1_ *y*, open probability of *I*_K1_ polyamine gate. (C) *I*_CaL_, L-type calcium current; *I*_CaL_ p(AP), open probability of *I*_CaL_ voltage-dependent gate; *I*_CaL_ p(U), open probability of *I*_CaL_ calcium gate; *I*_CaL_ *y*, open probability of *I*_CaL_ ultra-slow gate. (D) *I*_CaT_, T-type calcium current; *I*_CaT_ *y*_1_, open probability of *I*_CaT_ activation gate. (E) *I*_NaCa_, Na^+^/Ca^2+^ exchange current. (F) *I*_*l*(Ca)_, Ca^2+^-activated background cation current. (G) *I*_RyR_, ryanodine receptor channel current; *I*_RyR_ p(Open), open probability of *I*_RyR_ gate. (H) *I*_SERCA_, SR Ca^2+^ pump current; *I*_SERCA_ *y*, probability of the conformation state with the Ca^2+^-binding sites onto the SR side. (I) [Ca^2+^]_rel_, calcium concentration in SR release site.

**Fig 4:**
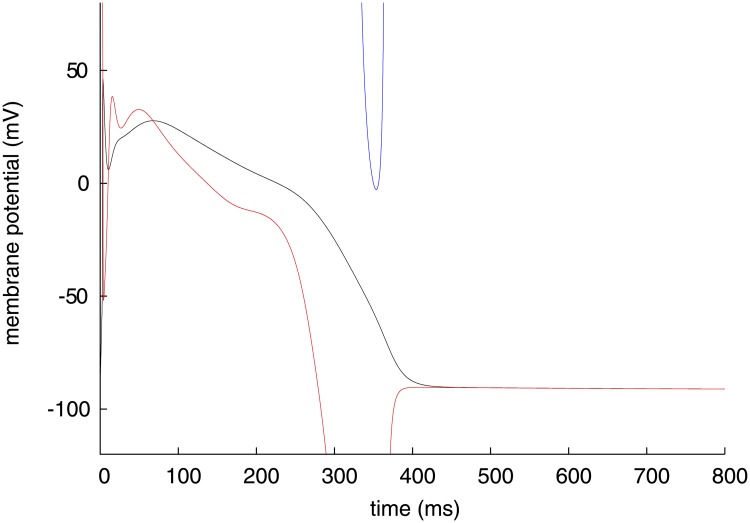
Time course of membrane potential (black) and its stable and unstable instantaneous equilibrium point (red and blue, respectively) of Priebe model.

**Fig 5:**
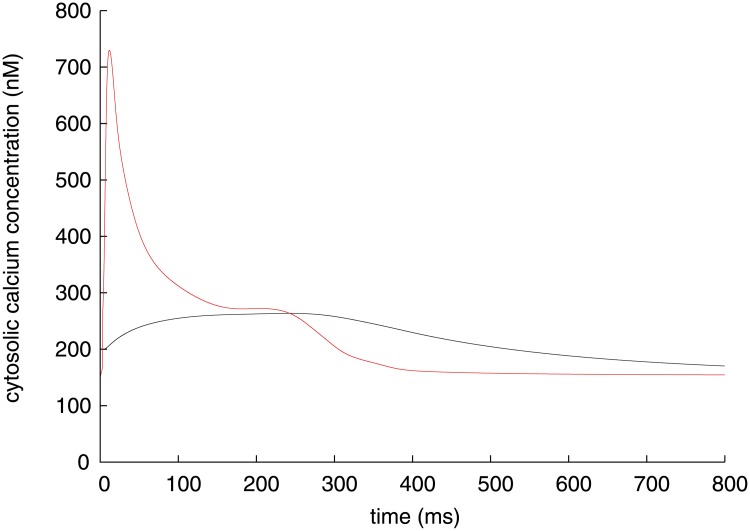
Time course of intracellular calcium concentration (black) and its instantaneous equilibrium point (red) of Priebe model.

In order to analyse the mechanisms of action potential generation and calcium transient, which are central functions in ventricular myocyte, object variables in the present analysis are the membrane potential and the intracellular calcium concentration. For the Takeuchi model, the amount of cytosolic calcium n(Ca)_i_ replaces the intracellular calcium concentration, because the concentration is not a differential variable but defined as n(Ca)_i_ divided by the cellular volume. For the Takeuchi model, simulated time courses of the membrane potential *V*_*m*_ and n(Ca)_i_ to be analysed are shown with black lines in Figs 1 and 2, respectively. The Takeuchi model reproduces a typical ventricular action potential with a long plateau, which is formed by inward and outward ion currents through cellular membrane. The major inward currents are *I*_Na_, *I*_CaL_, *I*_CaT_ and *I*_NaCa_, and the outward currents are *I*_K1_ and *I*_l(Ca)_. An intracellular calcium transient is determined in the Takeuchi model by transmembrane Ca^2+^ currents (*I*_CaL_, *I*_CaT_ and *I*_NaCa_) and Ca^2+^ fluxes across the sarcoplasmic reticulum (SR) membrane (*I*_RyR_ and *I*_SERCA_). These ion currents and fluxes are shown with black areas in Fig 3. Differential variables for open probabilities of channel gates, state variables of transporters, and calcium concentration in SR are also plotted with solid lines. In a similar manner, for the Priebe model, simulated time courses of the membrane potential *V* and the intracellular calcium concentration [Ca^2+^]_i_ are plotted with black lines in Figs 4 and 5, respectively. All the time courses of model differential variables are identical to those in [1] and [5].

### Dynamics of Action Potential

For the Takeuchi model, a red line in Fig 1 represents the instantaneous equilibrium point (IEP) of *V*_*m*_ at each time defined in Eq 4, where *V*_*m*_, ${\dot{V}}_{m}$ and the partial derivative of ${\dot{V}}_{m}$ function with respect to *V*_*m*_ are substituted for *x*, $\dot{x}$ and *f*_*x*_, respectively. Specifically, the IEP of *V*_*m*_, $\bar{V_{m}}$ is expressed as

$$\begin{array}{c} \bar{V_{m}}=V_{m}-{\dot{V}}_{m}/\frac{\partial {\dot{V}}_{m}}{\partial V_{m}}. \end{array}$$ (10)

**Fig 6:**
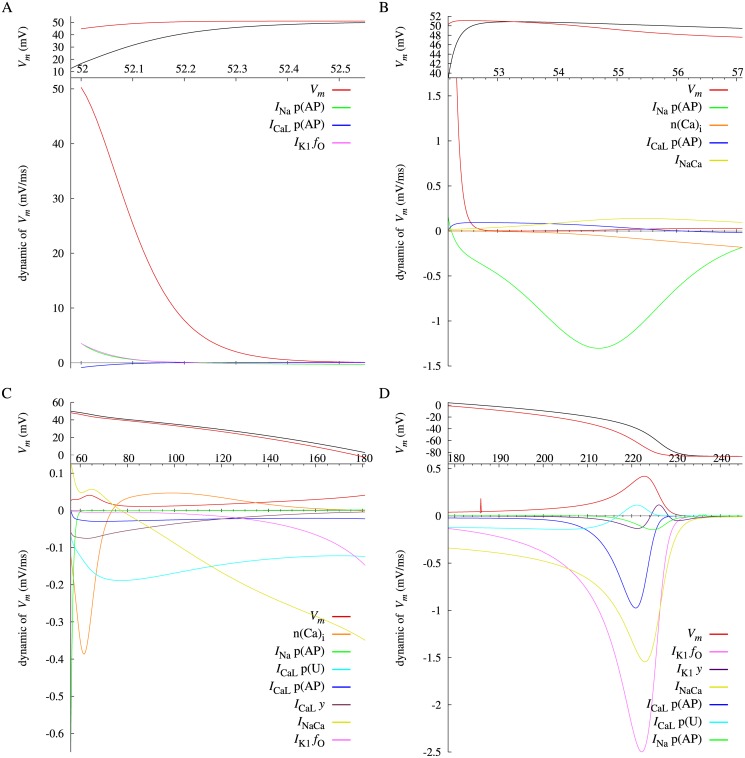
Dynamics of the membrane potential *V*_*m*_ of Takeuchi Model after stimulus offset at different phases (A–D) with different scales, together with *V*_*m*_ (black) and the instantaneous equilibrium point (red) at the top of each figure on the same time base. *I*_NaCa_ denotes the sum of *I*_NaCa_ p(E_1total_), p(I_1_) and p(I_2_) dynamics of *V*_*m*_. A notch of *V*_*m*_ dynamic around 185 ms and a tiny fluctuation of *I*_Na_ p(AP) dynamic around 235 ms in D are errors associated with division by nearly zero in numerical differentiation.

**Fig 7:**
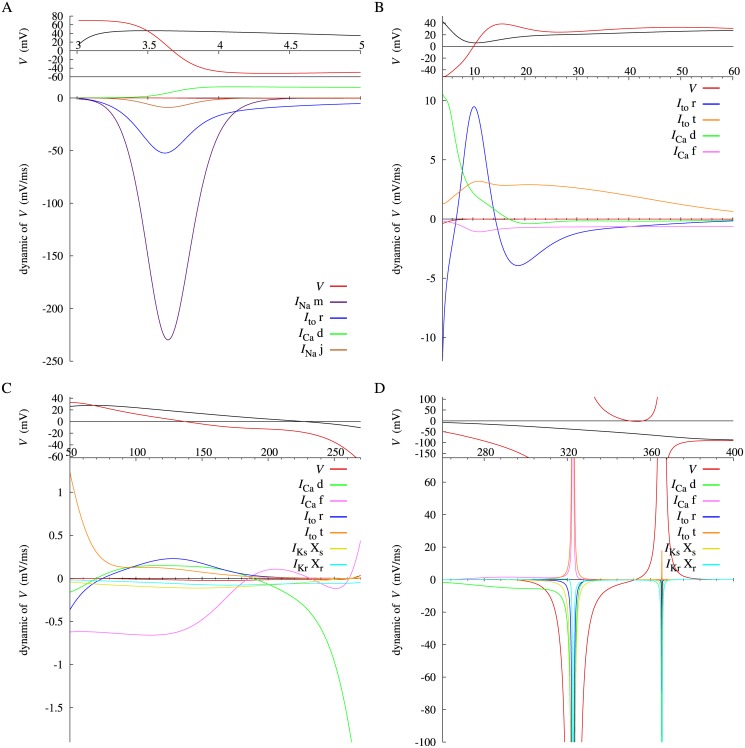
Dynamics of the membrane potential *V* of Priebe model after stimulus offset at different phases (A–D) with different scales, together with *V* (black) and the instantaneous equilibrium point (red) at the top of each figure on the same time base.

The IEP is higher than *V*_*m*_ during depolarization phase but lower during repolarization phase, that is, *V*_*m*_ is always attracted to its IEP, because the IEP is stable or *f*_*x*_ in Eq 5 is negative throughout the simulation period. The orbit of the IEP intersects with the time course of *V*_*m*_ exactly at the peak of *V*_*m*_ (∼ 53 ms) for Eq 5. Note that IEP during 50–52 ms is not effective to analysis due to the external current injection. Along with *V*_*m*_ time course and its IEP on the same time base in the upper panel, each variable dynamic of *V*_*m*_ with a significant value is plotted in the lower panels of Fig 6, separated into four sequential phases of the cardiac action potential; the last 0.5 ms of rising phase of the action potential after termination of the triggering current injection (phase 0, Fig 6A), the initial repolarizing phase of 4 ms (phase 1, Fig 6B), the major plateau phase (phase 2, Fig 6C), and the final repolarization (phase 3, Fig 6D). Note that, for a differential variable *v*, *v* dynamic of *V*_*m*_ is expressed as $\partial \bar{V_{m}}/\partial v\cdot dv/dt$ corresponding to Eq 7. Similarly for the Priebe model, a red line in Fig 4 represents the stable IEP of *V*, and a blue line during 323–365 ms represents the unstable IEP of *V*. Each variable dynamic of *V* with a significant value is plotted in the lower panels of Fig 7, separated into four sequential phases; the period including the *V* peak of 2 ms after termination of the triggering current injection (phase 0, Fig 7A), the initial repolarizing phase (phase 1, Fig 7B), the major plateau phase (phase 2, Fig 7C), and the final repolarization (phase 3, Fig 7D). IEP during 0–3 ms is not analysable due to the stimulation current.

An increase in the IEP of the membrane potential prompts an increase in the membrane potential, and vice versa. Therefore, a variable *v* induces a membrane depolarization if the *v* dynamic of *V*_*m*_ (or *V*) is positive, or a repolarization if negative. The sign of the *v* dynamic of *V*_*m*_ is determined by both *k*_*V*_*m*_,*v*_ = ∂*V*_*m*_/∂*v* and the derivative of *v*. Since an inward current itself induces an increase in *V*_*m*_, *k*_*V*_*m*_,*v*_ for an open probability of an inward current is positive. Therefore, a decrease in the open probability, or its negative derivative, induces a decrease in the IEP of *V*_*m*_ and contributes to repolarization, while an increase in the probability contributes to depolarization. For an outward current, the effect is opposite.

For the Takeuchi model, Fig 6A shows that the fast depolarization in phase 0 is exclusively attributed to the *V*_*m*_ dynamics of *V*_*m*_ itself. The details are explained later. In phase 1 shown in Fig 6B, *I*_Na_ p(AP), the open fraction of *I*_Na_ voltage gates is the dominant negative dynamic of *V*_*m*_. On the other hand, as shown in Fig 7A for the Priebe model, the IEP of *V* is already descending at the termination time of the stimulation current, and *V* simply increases towards the IEP. After the intersection of *V* and its IEP (∼ 3.5 ms), *V* decreases in pursuit of the IEP until 10 ms (Fig 7B). The main contributor to the decrease in the IEP is *m*, the activation gate of *I*_Na_ (Fig 7A). These results for both models agree with an accepted view that inactivation of *I*_Na_ voltage gate mainly causes the initial negative shift in *V*_*m*_. The reason for the large negative dynamic is that the open fraction is decreasing due to rapid inactivation while the reversal potential of *I*_Na_ is more positive than the IEP. For the Priebe model, *V* increases again after its initial negative shift, because the IEP exceeds *V* again (Fig 7B). The major contributors to the increase of the IEP are *r* and *t*, the activation gate and the inactivation gate of *I*_to_, respectively. This also agrees with a physiological view.

In phase 2 shown in Fig 6C for the Takeuchi model, the major contributors to membrane repolarization are *I*_CaL_ p(U) (open probability of *I*_CaL_ calcium gates), and n(Ca)_i_ subsequently replaced by *I*_NaCa_ (Na^+^/Ca^2+^ exchange current). *I*_CaL_ p(U) dynamic of *V*_*m*_ is negative for all phase 2 and least during 65–110 ms. Decreasing of *I*_CaL_ p(U) (Fig 3C) by gradual Ca^2+^-dependent inactivation results in a reduction of *I*_CaL_, which provides a significant inward current. This analysis shows explicitly that an inward current, which has been generally thought to induce depolarization, contributes to a repolarization when its open probability is decreasing. Two other variables in *I*_CaL_, voltage-dependent inactivation (*I*_CaL_ p(AP)) and ultra-slow inactivation (*I*_CaL_ *y*), also have negative dynamic values. Therefore, closing of *I*_CaL_ gates induces the repolarization in phase 2. From the opposite side, this is consistent with a physiological view that *I*_CaL_ has major contribution to forming the plateau phase of action potential. The same is true for the Priebe model. As shown in Fig 7C, *f*, the inactivation gate of *I*_Ca_ has negative and least dynamic of *V* until 175 ms. For the Takeuchi model, the n(Ca)_i_ dynamic of *V*_*m*_ is least during the early period of the phase 2 about 58–66 ms, but becomes positive after the peak of n(Ca)_i_ shown in Fig 2. These indicate that the rise in n(Ca)_i_ has a large repolarizing effect on the Takeuchi model. It is mainly attributable to an activation of *I*_l(Ca)_ (Fig 3F). Since *I*_l(Ca)_ is defined as directly depending on *V*_*m*_ and n(Ca)_i_ without any state variables in the model, its effect on the dynamics of *V*_*m*_ is not explicitly shown, and included in n(Ca)_i_ dynamics of *V*_*m*_ in this method. In Fig 7C for the Priebe model, *X*_*s*_ and *X*_*r*_, the activation gate of *I*_Ks_ and *I*_Kr_ respectively, have relatively small negative dynamics of *V*. In Fig 6C for the Takeuchi model, dynamics of *V*_*m*_ relating to *I*_Ks_ or *I*_Kr_ are not shown, because they are negligibly small. These results indicate that *I*_Ks_ and *I*_Kr_ make effects on the level of membrane potential in phase 2 but rather small dynamic effects to induce repolarization.

In the classical view, final repolarization towards the resting potential in phase 3 is mainly attributable to increase in *I*_K1_, decrease in *I*_CaL_, and deactivation of *I*_NaCa_. The results in Fig 6D show general agreement with the view, and give additional insights into the Takeuchi model. Voltage-dependent removal of Mg^2+^ block of *I*_K1_ (*I*_K1_ *f*_O_) induces the repolarization, whereas closing of voltage-dependent gates (*I*_K1_ *y*) prevent it slightly at late phase 3. Similarly, deactivation of *I*_CaL_ voltage gates (*I*_CaL_ p(AP)) induces the repolarization and opening of Ca^2+^-dependent gates (*I*_CaL_ p(U)) hinders it. Finally, the negative *I*_NaCa_ dynamic of *V*_*m*_ indicates a repolarizing effect of *I*_NaCa_, contrary to the fact that its inward current is increasing until ∼ 225 ms (Fig 3E). The negative *I*_NaCa_ dynamic, which denotes the sum of *I*_NaCa_ p(E_1total_), p(I_1_) and p(I_2_) dynamics, results from a rearrangement of molecular states of the exchanger to decelerate the exchange of intracellular Ca^2+^ for extracellular Na^+^. On the other hand, an inward current of *I*_NaCa_ is determined not only by the molecular states but also by driving force of Ca^2+^ influx through the exchanger, which is enlarged by membrane repolarization. Thus, *I*_NaCa_ affects the repolarization and is affected by it at the same time. The present method can discriminate the former active effect from the latter passive effect (see Discussion).

**Fig 8:**
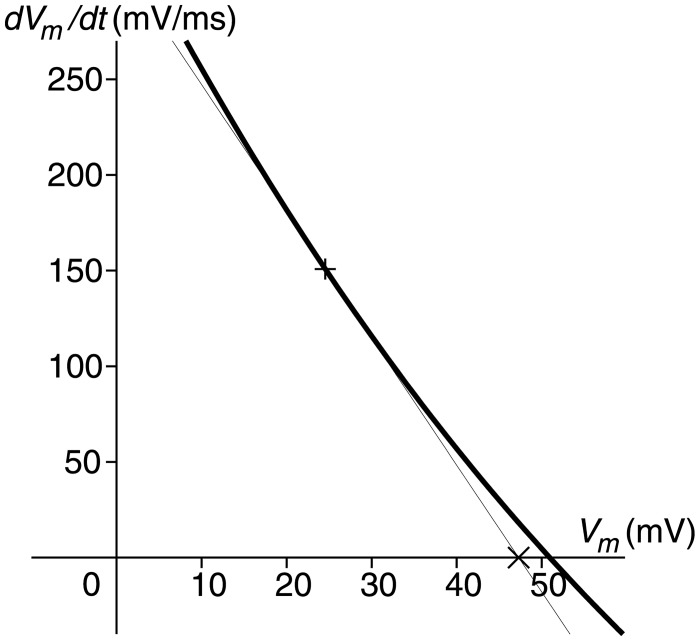
Derivative function of *V*_*m*_ of Takeuchi model at 52.05 ms (thick curve) assuming that all the other differential variables are constant. × is the instantaneous equilibrium point of *V*_*m*_, which is the x-intercept of the tangent line (thin line) at the value of *V*_*m*_ at that time (+).

**Fig 9:**
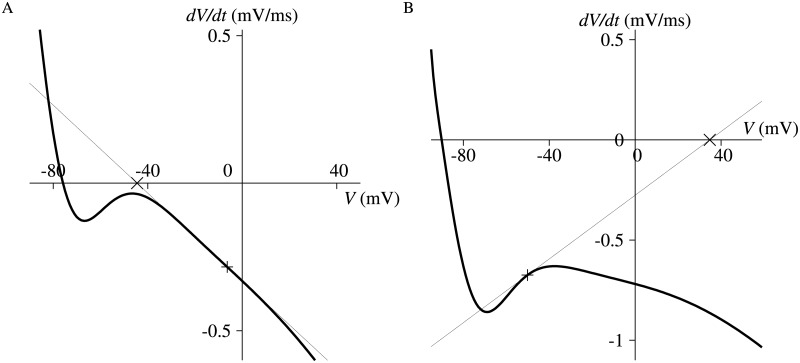
Derivative function of *V* of Priebe model at 257 ms and 340 ms (thick curve in A and B, respectively) assuming that all the other differential variables are constant. × is the instantaneous equilibrium point of *V*, which is the x-intercept of the tangent line (thin line) at the value of *V* at that time (+).

As shown in Fig 6A for the Takeuchi model, the *V*_*m*_ dynamic of *V*_*m*_ itself is dominant in phase 0. It indicates that the increase in *V*_*m*_ induces further depolarization. In this period, massive *I*_Na_, which is formulated with Goldman-Hodgkin-Katz flux equation, gives high nonlinearity to the derivative function of *V*_*m*_. For an instance, the derivative of *V*_*m*_ at 52.05 ms is shown in Fig 8. Due to the nonlinearity, a positive change in *V*_*m*_ simply shifts the x-intercept, or the IEP of *V*_*m*_, in the positive direction. In Fig 7D for the Priebe model, the *V* dynamic of *V* itself is dominant after 310 ms of the final repolarization phase. Fig 9 shows the derivatives of *V* at 257 ms and 340 ms. When *V* is greater than the local maximum of the derivative (Fig 9A), *V* is headed for its stable IEP, and a negative perturbation to *V* makes the IEP less because of the nonlinearity of the derivative. The IEP approaches to infinity as *V* gets close to the local maximum. When *V* exists between the local maximum and minimum (Fig 9B), *V* tends to move in the opposite direction of its unstable IEP, and a negative change in *V* shifts its IEP in the negative direction. Therefore, a reduction in *V* decreases the IEP, and this induces repolarization automatically in this phase. Note that enormous negative and positive dynamics of *V* balances around switching points of the stability of the IEP of *V*, because the derivative of the IEP is extremely large (Eq 9).

### Dynamics of Calcium Transient

**Fig 10:**
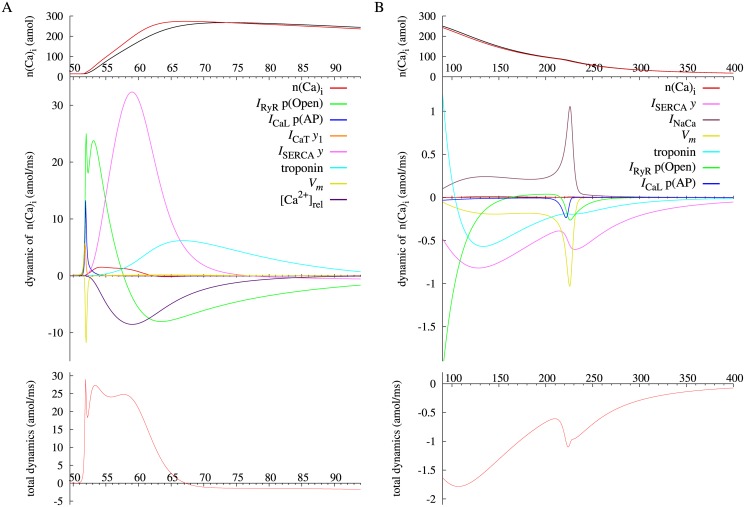
Each dynamic and total dynamics of amount of cytosolic calcium, n(Ca)_i_ of Takeuchi model after stimulus onset at two phases (A, B) with different scales, together with n(Ca)_i_ (black) and the instantaneous equilibrium point (red) at the top of each figure. *I*_NaCa_ stands for the sum of *I*_NaCa_ p(E_1total_), p(I_1_) and p(I_2_) dynamics of n(Ca)_i_, and troponin stands for the sum of [T], [TCa], and [TCa*] dynamics of n(Ca)_i_.

**Fig 11:**
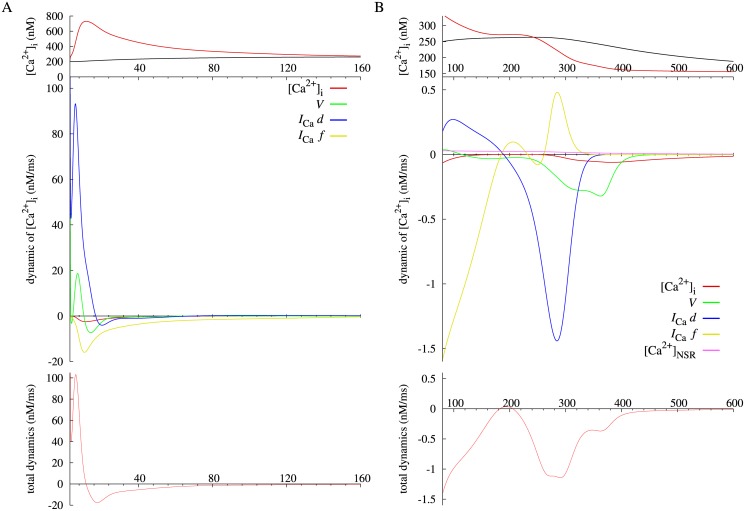
Each dynamic and total dynamics of intracellular calcium concentration, [Ca^2+^]_i_ of Priebe model after stimulus onset at two phases (A, B) with different scales, together with [Ca^2+^]_i_ (black) and the instantaneous equilibrium point (red) at the top of each figure.

Another practice of the present method is on calcium transient, which is a transient elevation of intracellular Ca^2+^ concentration. For the Takeuchi model, the amount of cytosolic calcium n(Ca)_i_ and its IEP are plotted with a black and red lines in Fig 2, respectively. The IEP leads the time course of n(Ca)_i_ because the IEP is stable throughout the simulation period. In the Takeuchi model, the calcium transient is determined by balance among three mechanisms; (1) the net Ca^2+^ flux across the surface membrane via L-type and T-type Ca^2+^ channels (*I*_CaL_, *I*_CaT_), Na^+^/Ca^2+^ exchangers (*I*_NaCa_) and plasma membrane calcium pumps (*I*_PMCA_), (2) the net Ca^2+^ flux across the SR membrane via ryanodine receptors (*I*_RyR_) and calcium pumps (*I*_SERCA_), and (3) Ca^2+^-binding to cytosolic Ca^2+^ buffer proteins. For measuring contributions of these factors, each variable dynamic of n(Ca)_i_ with a significant value are plotted along with total dynamics, the sum of all the dynamics, in two periods; rising and plateau phases of n(Ca)_i_ in Fig 10A, and falling phase in Fig 10B. The total dynamics of n(Ca)_i_ is equivalent to the derivative of the IEP of n(Ca)_i_ for Eq 9. For the Priebe model also, intracellular calcium concentration [Ca^2+^]_i_ and its stable IEP are plotted with a black and red lines in Fig 5, respectively. Individual dynamics of [Ca^2+^]_i_ and the total dynamics are shown in two phases; upward phase of [Ca^2+^]_i_ in Fig 11A, and downward phase in Fig 11B. Similarly to the analysis of the membrane potential, a positive dynamic of n(Ca)_i_ or [Ca^2+^]_i_ indicates an effect to vary the IEP in the positive direction, and vice versa.

In the general physiological view, the rising phase of the cytosolic calcium level is attributable to calcium-induced calcium release (CICR), in which a slight increase in intracellular Ca^2+^ by the activation of *I*_CaL_ and *I*_CaT_ further triggers a massive Ca^2+^ release from sarcoplasmic reticula into cytosol through ryanodine receptors (RyRs). In Fig 10A for the Takeuchi model, the total dynamics of n(Ca)_i_ has the first very brief positive peak during rapid depolarization of the cellular membrane. This peak is formed by *I*_CaL_ p(AP) dynamic and *I*_CaT_ *y*_1_ dynamic of n(Ca)_i_ and additionally by the first sharp peak of *I*_RyR_ p(Open) dynamic, which is caused by an activation of RyRs proportional to Ca^2+^ flux through *I*_CaL_. These positive dynamics drive n(Ca)_i_ in the positive direction slightly. The second wave of the total dynamics is mainly attributed to *I*_RyR_ p(Open) via an increase in cytosolic Ca^2+^ itself caused by the first peak of the total dynamics. These analysis results of the Takeuchi model are in good agreement with the view of the CICR mechanism. However, subsequent results reveal an important difference. *I*_RyR_ p(Open) begins to decrease at 57.8 ms (Fig 3G) during the rise in the IEP of n(Ca)_i_ due to time-dependent closure of RyRs. This decrease makes *I*_RyR_ p(Open) dynamic negative and hinders the rise in n(Ca)_i_. In addition, the Ca^2+^ release via RyRs rapidly decreases [Ca^2+^]_rel_, total calcium concentration in SR release site (Fig 3I). This decrease also hampers the increase in n(Ca)_i_ via reduction in the Ca^2+^ concentration gradient across the SR membrane to drive *I*_RyR_, as indicated by negative [Ca^2+^]_rel_ dynamic in Fig 10A. Alternatively, the prominent contributor in the Tekeuchi model to the increase in the IEP of n(Ca)_i_ after 55 ms is *I*_SERCA_, which is to reduce n(Ca)_i_ by pumping cytosolic Ca^2+^ into SR though. In a rough explanation, deactivation of *I*_SERCA_ decreases Ca^2+^ flux from cytosol to SR and so induce to increase n(Ca)_i_. To be precise, *I*_SERCA_ *y* is the fraction of carrier proteins exposing their Ca^2+^-binding sites toward SR lumens and negatively represents a capability of *I*_SERCA_ to transfer calcium ions from cytosol. *I*_SERCA_ *y* dynamic is positive after 52 ms until 77 ms because of the increase in *I*_SERCA_ *y*, although *I*_SERCA_ is increasing until 56 ms and decreasing still after 77ms (Fig 3H) due to passive dependency of *I*_SERCA_ on cytosolic Ca^2+^ concentration. Additionally, the results show that troponin, which is a cytoplasmic calcium buffer protein, partially contributes to the rise in the IEP of n(Ca)_i_ until 104 ms (Fig 10A and 10B) by a decrease in concentration of calcium-free troponin, or Ca^2+^ binding sites.

The negative total dynamics of n(Ca)_i_ shown in Fig 10B facilitates the last calcium decrease. *I*_RyR_ p(Open) dynamic is continuously negative since the late rising phase until 162 ms. Additionally, negative *I*_SERCA_ *y* dynamic via a decline in *I*_SERCA_ *y* and negative troponin dynamic by the opposite of the aforementioned effect account for a large portion of the total dynamics of n(Ca)_i_. In contrast, *I*_NaCa_ dynamic of n(Ca)_i_ is positive due to increased proportion of inactive conformation states of the exchanger, whereas an inward current of *I*_NaCa_ (Fig 3E) to carry Ca^2+^ out of cell is increasing until the end of action potential (∼ 225 ms) because of an increase in the voltage-dependent transport rate of *I*_NaCa_. An effect of the voltage-dependency on n(Ca)_i_ is represented by a major part of negative *V*_*m*_ dynamic. Therefore, overall contribution of *I*_NaCa_ to dynamic changes of cytosolic calcium level is relatively small.

For the Priebe model, the mechanism of calcium transient is rather simple, because the calcium release and uptake of SR and *I*_NaCa_ are passively formulated without differential variables. As shown in Fig 11, the elevation of [Ca^2+^]_i_ triggerd by membrane depolarization is attributed to *d*, the activation gate of *I*_Ca_ and partially *V*, as is the case in the Takeuchi model. Although the IEP of [Ca^2+^]_i_ descends after 12 ms caused by the deactivation of *I*_Ca_ (*f*), the IEP is sufficiently high to raise [Ca^2+^]_i_. The time course of the IEP has a plateau around 200 ms, because *d* and *f* dynamics of [Ca^2+^]_i_ come to zero and balance with each other. Finally, the main contributors to a decrease in [Ca^2+^]_i_ are closing of the activation gate of *I*_Ca_ (*d*) and voltage dependency of the transmembrane calcium flux (*V*).

### Summary of Analyses

Analysis results in Figs 6 and 7 confirm that the Takeuchi model and the Priebe model provide generally good agreement with the classic qualitative view on the ionic mechanisms of the action potential generation in ventricular myocytes. On the other hand, these analyses exhibit that inward currents can contribute to the repolarization via their decrease, in contrast to a classic physiological concept. In the plateau phase, inactivation of Ca^2+^ channels prompts the repolarization. Additionally, these analyses provide quantitative insights into the contribution of each gating mechanism and ion exchanger activity. For instance, *I*_Ks_ and *I*_Kr_ have only small dynamic effects to induce membrane repolarization. In the last phase of membrane repolarization, Mg^2+^ block and voltage-dependent gates of *I*_K1_ contradictorily affect the repolarization. These insights into the dynamic mechanisms of the action potential generation will be useful for controlling the action potential.

Analyses on calcium transient of the Takeuchi model show good agreement with the general physiological view of the CICR mechanism in the early phase of [Ca^2+^]_i_ elevation, but also provide an important difference. [Ca^2+^]_i_ rising in the late part is attributable to the reduced capability of SERCA to take calcium ions into SR. The analyses results also show that the decay phase of calcium transient on the Takeuchi model is attributable to decrease in calcium release from SR through *I*_RyR_ initially and to *I*_SERCA_ and troponin thereafter.

## Discussion

### Feature of the Proposed Method

This article presents a novel analytical method, which is applicable to a complicated mathematical cell model with many variables, for decomposing and quantifying contributions of individual variables to dynamic change in a variable of interest at each moment during simulations. Toward this end, the method employs a representative point of the instantaneous trend in the object variable, ‘instantaneous equilibrium point’, in which the temporal change characterises dynamics of the object variable. The contributions of variables are evaluated with a quantitative index, ‘dynamic of the object variable’, of which value is a variable component of a variation in the instantaneous equilibrium point. Applications of the present method to a ventricular myocyte model demonstrate its capability to mathematically quantify dynamic interactions among variables of a mathematical model and thereby explicate principle of the model.

### Comparison with Other Methods

A conventional method applied to mathematical cell models is sensitivity analysis. In a typical application manner of sensitivity analysis, variations of simulated results are observed against variations of model parameters. This analysis is useful for examining comprehensive influence of chemical or genetic modifications to cellular components. However, estimating contributions of cellular components to cellular dynamic behaviour is difficult by using this analysis, because a modification to a model parameter causes cascade effects on cellular dynamics. For an instance, a decrease in a parameter for the amplitude of Ca^2+^ current results in not only a decrease in the Ca^2+^ current but also a certain decrease in calcium level, and the decrease in calcium level affects Ca^2+^-dependent processes, which make further effects. In contrast, the present method is able to quantify contributions of cellular components to cellular dynamics in the intact situation. This is a distinguishing feature of the present method.

A method developed to analyze contribution of current components to an action potential is ‘lead potential analysis’ proposed by Cha et al [19]. The present method can be regarded as a generalized version of the lead potential analysis. The *lead potential* (*V*_*L*_) in the Cha’s method [19, 20] is almost equivalent to the instantaneous equilibrium point of the membrane potential, and *contribution* (*c*_*i*_) or *relative contribution* (*r*_*c*_) corresponds to the dynamic of the membrane potential. The results in Fig 6 of this article are similar to their analysis results of the Takeuchi model in Fig. 5 in [19]. Compared to the lead potential analysis, the present method has improvements and advantages in several aspects. Firstly, the *c*_*i*_ or *r*_*c*_ in Cha’s method is procedurally defined, in contrast to the mathematically well-defined present method. Next, the lead potential analysis has a limitation on treatment of ion transporters as discussed in [19]. To calculate the lead potential, the membrane current system through ion channels and transporters are approximately described with an equivalent electrical circuit. While each channel current is straightforwardly expressed in the circuit as a pair of a battery for the corresponding equilibrium potential and a variable conductance to express its dynamic gating, ion transporters are approximated as current sources [19, 21, 22] or ohmic channels [20] depending on the studies. As a result of the approximation, the contribution of a transporter includes not only effect of its activity on the membrane potential but also passive effect of the membrane potential on the transporter. Since the present method mathematically properly processes all components in the same manner, a dynamic of the membrane potential only expresses active effect. For instance, the dynamic effect of conformational changes of *I*_NaCa_ on membrane potential is successfully discriminated in *I*_NaCa_ dynamic of *V*_*m*_ in Fig 6, in contrast to *r*_*c*_ of *I*_NaCa_ in Fig. 5 in [19]. Finally, in the lead potential analysis, selection of current components of which *r*_*c*_ are to be calculated is arbitrary and demanding, because the components should be selected so that the summation of *r*_*c*_ of all the selected components is exactly 1. In the present method, a set of variables whose dynamic are calculated is automatically and uniquely determined on the set of all differential variables. For instance, *I*_l(Ca)_ dynamic is not defined in the application of the present method to the Takeuchi model because *I*_l(Ca)_ is formulated with no state variable, whereas results in [19] include the contribution of *I*_l(Ca)_ to the membrane potential.

Another numerical method for analysis of action potential dynamics is ‘dominant scale method’ proposed by Clewley et al [17]. with the main aim of model reduction. This method introduces the *instantaneous asymptotic target*, *x*_∞_ such that *f*(*x*_∞_, **y** ^(*t*_*c*_)^) = 0 at *t*_*c*_ for $\dot{x}=f(x,\text{y})$, or a fixed point of Eq 2 in the present article. Then, ∂*x*_∞_/∂*y* is utilized as an index of dominance, and ∣(∂*V*_∞_/∂*s*)(*ds*/*dt*)∣ is referred to as the *instantaneous rate of influence* [18], where *V* is the membrane potential and *s* is a gating variable. The dominant scale method is identical to the application of the present method to the membrane potential if *dV*/*dt* is linear with regard to *V*, as in the case of Hodgkin-Huxley model shown in [18]. The primary difference of the present method from the dominant scale method is linearization of the derivative function around the current values of the object variable. Whereas the instantaneous asymptotic target is a global fixed point of a system, the instantaneous equilibrium point is to represent the instantaneous trend in an object variable as described in Method section. This is an advantage of the present method. Fig 9A displays a situation where the instantaneous asymptotic target, which is the intersection of the derivative curve and the x-axis (∼ −75 mV), is far from the instantaneous equilibrium point (∼ −44 mV). Although the derivative is zero at the instantaneous asymptotic target, the direction that *V* is heading in at that moment is the instantaneous equilibrium point. Another advantage of the present method is the uniqueness of the instantaneous equilibrium point. There always exists a unique instantaneous equilibrium point of an object variable of a system except if *f*_*x*_ is exactly equal to zero, whereas there can exist no or multiple instantaneous asymptotic targets. In fact, the derivative of *V* of the Priebe model has three instantaneous asymptotic targets in the plateau phase.

### Limitations and Applications

In the present method, objects of analysis are limited to differential variables. This property is both an advantage and a disadvantage. Since the dynamics of an object variable are not defined with respect to variables of which values are passively determined by other variables, passive components are automatically excluded in analysis. However, in some models probabilities of gate opening and conformation states are not formulated as differential equations but as regular equations assuming instantaneous equilibria for a reason such as computational simplicity or numerical stability. In order to consider the corresponding dynamic effects in such cases, model equations require to be transformed into differential equations in the same manner as *I*_K1_ *f*_*O*_ of the Takeuchi model.

The present method is applicable to recent and complicated ventricular myocyte models [23, 24], although rather simple models analysed in this paper are suitable for the initial demonstration of the proposed method. Moreover, many computational models have been published for various physiological phenomena such as electrical activity, signal transduction, and metabolism. Applications of the present method are not limited to cardiac cell models but possible to any kind of models defined as a system of ordinary differential equations. On the other hand, the present method is not applicable to models that utilize formalisms other than ODE, such as cardiac cell models including detailed spatial-temporal expressions of calcium cycling.

The present method will be useful for analyses of transient phenomena and oscillatory phenomena such as afterdepolarizations of cardiac myocytes, because individual dynamics can be evaluated at any instant of a process. Control of arrhythmia is a potential application of the present analysis approach. Furthermore, the present method can analyse dynamics of a variable that contributes to dynamics of another variable successively. To take the application to the Takeuchi model as an instance, since n(Ca)_i_ has a primary effect on decrease in *V*_*m*_ during the rising phase of n(Ca)_i_ and *I*_SERCA_ *y* is the main contributor to the rise in n(Ca)_i_, it is quantitatively clarified that the activity of *I*_SERCA_ indirectly affects the membrane repolarization. Such consecutive analyses will help quantitative explanations of pathways of physiological phenomena.

## Supporting Information

S1 Appendix Modified I_K1_ Magnesium Blockade Model. (PDF)
