# Supplementary material for: Quantitative Decomposition of Dynamics of Mathematical Cell Models: Method and Application to Ventricular Myocyte Models
Source: PLoS One. 2015 Jun 19;10(6):e0124970. doi: 10.1371/journal.pone.0124970 (PMC4474442; doi:10.1371/journal.pone.0124970)
Supplement: S1 Appendix — (PDF) [file pone.0124970.s001.pdf]

Modified I_{K1} Magnesium Blockade Model

In a ventricular model by Takeuchi et al.[1], open probability of magnesium block of I_{K1} is defined with a steady-state expression as

$$f_{\mathrm{O}} = \frac{\lambda}{\mu + \lambda},$$

where λ and μ are rate constants depending on the membrane potential.

For analysis with taking into account effects of magnesium blockade of I_{K1}, the above equation is replaced by the following equation.

$$\frac{df_{\mathrm{O}}}{dt} = \lambda\,(1 - f_{\mathrm{O}}) - \mu f_{\mathrm{O}}.$$

References

[1] Takeuchi A, Tatsumi S, Sarai N, Terashima K, Matsuoka S, Noma A. Ionic Mechanisms of Cardiac Cell Swelling Induced by Blocking Na^{+}/K^{+} Pump As Revealed by Experiments and Simulation. Journal of General Physiology. 2006 Nov;128(5):495–507.
